# Regularization via gradient attribution for multiple sclerosis lesion segmentation

**DOI:** 10.3389/fmed.2026.1907567

**Published:** 2026-09-23

**Authors:** Salvatore Giugliano, Giovanna Sannino

**Affiliations:** Institute for High-Performance Computing and Networking (ICAR), National Research Council of Italy (CNR), Naples, Italy

**Keywords:** deep learning, explainable artificial intelligence, internet of medical things, loss function, multiple sclerosis, precision medicine, segmentation

## Abstract

**Introduction:**

Accurate segmentation of Multiple Sclerosis (MS) lesions from Magnetic Resonance Imaging (MRI) is fundamental for robust disease monitoring and for the evaluation of therapeutic efficacy. Within Internet of Medical Things (IoMT)-based diagnostic infrastructures, where deep learning models serve as autonomous computational nodes embedded in interconnected clinical workflows, ensuring reliable lesion detection without continuous human supervision is a critical operational and clinical requirement. A persistent methodological challenge is the pronounced class imbalance characteristic of MS neuroimaging datasets. This imbalance causes conventional loss functions to systematically miss small lesions, thereby directly compromising the utility of automated image analysis as an instrument for precision medicine.

**Methods:**

This work proposes the Regularization via Gradient Attribution (RGA) framework, a module that leverages gradient-based eXplainable Artificial Intelligence (XAI) attribution maps as active supervisory signals during training. Instead of constraining the learning process exclusively at the prediction-output level, RGA imposes additional penalties on the network when false-negative lesion regions have low internal attribution and when false-positive healthy-tissue regions exhibit elevated attribution. The framework is architecturally agnostic, compatible with both convolutional and transformer-based backbone networks, and accommodating arbitrary spatial attribution techniques. RGA was evaluated on two public MS lesion segmentation datasets, MSLesSeg and 3D-MR-MS, using three backbones (nnU-Net, UNETR, TransBTS) and two XAI methods (LayerCAM, Integrated Gradients) in 5-fold cross-validation.

**Results:**

On both datasets, RGA consistently improved DSC, TPR, and LTPR over the baselines. The best configuration, nnU-Net + RGA achieved a DSC of 0.7056 on MSLesSeg and 0.7466 on 3D-MR-MS, with RGA yielding the highest DSC for all backbones. Qualitative analyses further substantiate these findings by demonstrating a marked reduction in missed-lesion regions across all model architectures, without a corresponding substantial increase in false-positive predictions.

**Discussion:**

By jointly providing high lesion-level detection completeness, model interpretability, and zero additional inference-time computational cost, RGA represents a methodologically grounded approach compatible with IoMT-oriented precision-medicine pipelines, while requiring dedicated deployment validation before clinical or edge-device implementation.

## 1 Introduction

Multiple Sclerosis (MS) is a chronic autoimmune and neurodegenerative disorder of the central nervous system, affecting approximately 2.8 million individuals worldwide and representing the leading cause of non-traumatic neurological disability in young adults (1). It is characterized by inflammatory demyelination and axonal injury, resulting in the progressive accumulation of focal white matter lesions (plaques) and, to a lesser extent, diffuse grey matter pathology. This chronic and heterogeneous course demands sustained clinical surveillance to track lesion burden dynamics and therapeutic response over time. The rise of connected health platforms and Internet of Medical Things (IoMT) technologies (2) has further amplified the feasibility of such long-term monitoring, enabling remote data acquisition and the integration of longitudinal patient records into intelligent diagnostic pipelines. Within this evolving landscape, Magnetic Resonance Imaging (MRI) remains the gold-standard modality for in vivo diagnosis and follow-up assessment of MS (3). Accurate and reproducible quantification of lesion count, volume, and spatial distribution is thus indispensable for objectively gauging disease progression and evaluating treatment response (4).

Historically, the segmentation of MS lesions has relied on manual annotation by trained neuroradiologists, a workflow that is both labor-intensive and subject to substantial intra- and inter-rater variability (5). Automated solutions based on Artificial Intelligence (AI) and Deep Learning (DL), in particular 3D Convolutional Neural Networks (CNNs) have consequently risen to prominence as the reference approach for fully automated MS lesion segmentation (6, 7). In the broader context of IoT-driven healthcare (8), these automated segmentation modules serve as the core computational nodes in remote diagnostic pipelines, enabling scalable, asynchronous analysis of MRI data without requiring on-site expert intervention.

Despite their overall strong performance, DL-based segmentation models face inherent challenges when applied to MS data: lesions are frequently sub-centimeter in size, display ill-defined boundaries, and constitute only a marginal fraction of the total brain volume, typically between 1% and 5% (9). This pronounced class imbalance drives conventional objective functions, such as Dice loss (10) and Cross-Entropy (CE) (11), to disproportionately emphasize global volumetric accuracy. As a result, the models are more prone to generating False Negatives (FNs), as they tend to miss small or unusual plaques. (12).

Concurrently, growing concerns about the opacity of deep neural networks have underscored the urgent need for trustworthy and clinically interpretable AI (13), a requirement that becomes particularly pressing when DL models are embedded as automated decision nodes within IoT healthcare architectures (14). eXplainable Artificial Intelligence (XAI) methods based on gradients or activations address this concern by producing dense spatial attribution maps that expose the image regions driving a given network prediction. A nascent research direction has begun repurposing these attribution maps beyond post-hoc inspection: rather than deploying XAI solely as a post-hoc diagnostic instrument, recent studies have demonstrated the feasibility of incorporating attention maps directly into the training objective (15–18). Extending this paradigm to 3D medical image segmentation, however, remains largely unexplored. In MS lesion segmentation specifically, XAI is still primarily applied for post-hoc visual interpretation (19), with no mechanism to actively steer the model’s internal focus during optimization.

To the best of our knowledge, no prior work has investigated the use of XAI as a direct training-time supervisory signal to spatially align network attention with ground-truth lesion annotations in the setting of 3D volumetric medical image segmentation. Whereas conventional loss functions operate solely at the level of the final prediction and existing attribution-guided regularization methods have been confined to 2D image classification tasks (15), the approach proposed here generalizes this paradigm to volumetric segmentation. Specifically, we introduce explicit regularization terms that penalize both insufficient attention to pathological regions and spurious activations within healthy tissue, thereby enforcing a joint optimization of predictive performance and the model’s internal decision-making process. Such spatial alignment of network attention is particularly advantageous in scalable IoT-based pipelines, where model reliability must be ensured without continuous human supervision.

In MS segmentation, FNs carry greater clinical significance than False Positives (FPs): an undetected lesion directly distorts the assessment of disease burden and progression, whereas a spurious detection is typically correctable during radiological review. This asymmetry becomes even more critical in remote monitoring scenarios enabled by IoMT platforms (2, 8), where timely clinical follow-up may be delayed and automated analysis constitutes the primary diagnostic output. Motivated by post-hoc attribution observations linking missed lesions to markedly attenuated internal activations, this work proposes elevating XAI from a reactive inspection tool to a proactive training-time regularization signal. The resulting *Regularization via Gradient Attribution (RGA)* framework penalizes the network when FN regions exhibit low attribution and when FP regions display unwarranted attention. The RGA framework is conceived as a regularization module, architecturally agnostic by design: it is compatible with heterogeneous segmentation backbones, from conventional CNNs to Vision Transformers (ViTs) (20), and supports any XAI attribution method that yields a dense spatial importance map. From a computational standpoint, the XAI-guided regularization scheme incurs no additional computational overhead during inference. The computation of attribution maps and the application of gradient-attribution penalties are restricted exclusively to the training phase, leaving the network’s inference pipeline entirely unchanged. This characteristic is of direct practical significance for IoMT deployments, in which integrating deep learning–based inference into resource-constrained edge devices is a primary technical challenge (21, 22). However, the present study does not include an end-to-end IoMT deployment or an edge-device benchmark.

Figure 1 schematically depicts the core principle of RGA: the exploitation of XAI-derived attribution maps as an additional, training-time supervisory signal to enforce coherence between the network’s internal attention patterns and the ground-truth lesion annotations. Regions corresponding to lesions that receive insufficient attention, as well as non-pathological regions that are spuriously over-attended, are converted into regularization terms that are incorporated into the optimization objective alongside the primary segmentation loss. A detailed formalization of this procedure is provided in Section 3.5.

**Figure 1:**
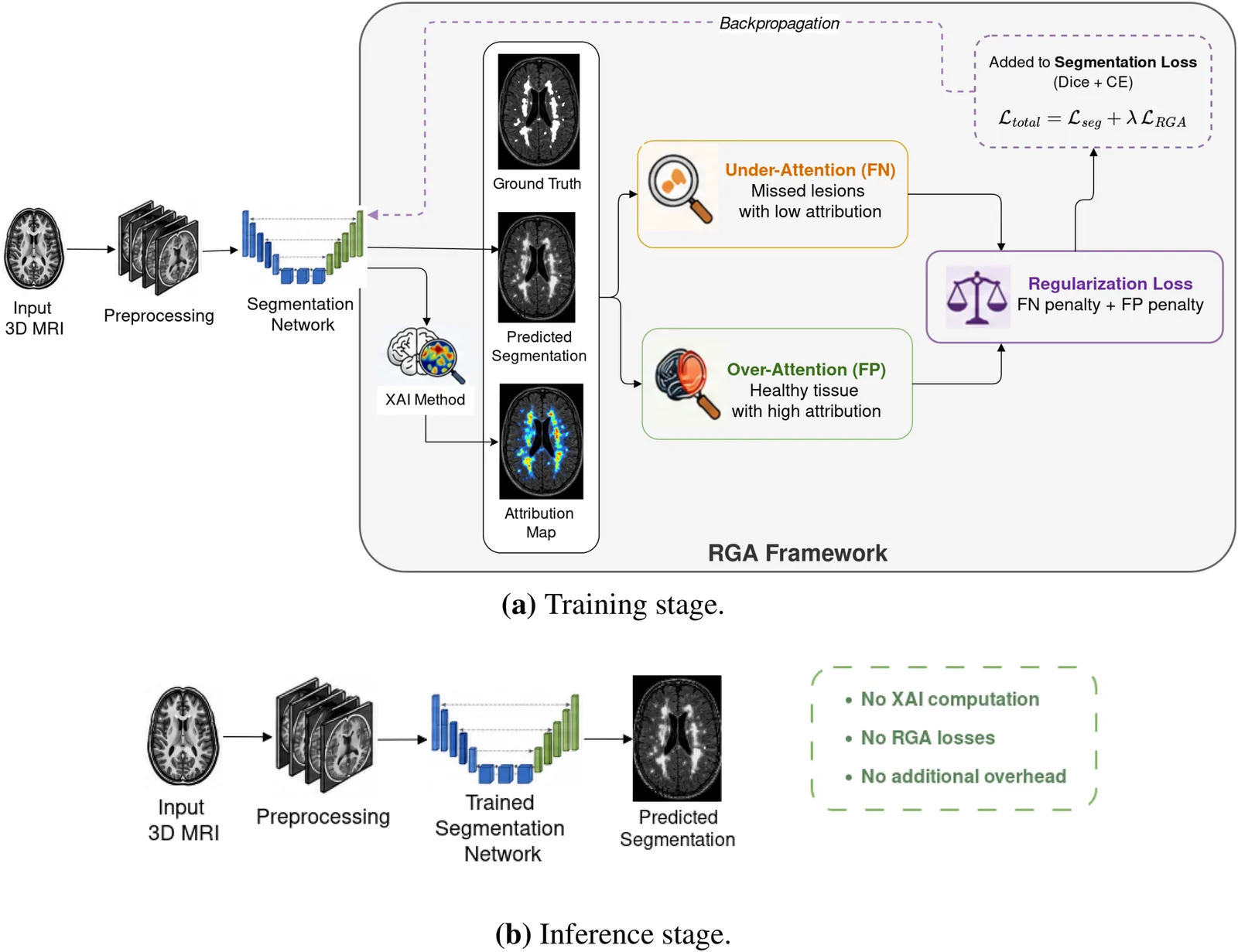
Overview of the proposed Regularization via Gradient Attribution (RGA) framework. Panel **(a)** shows the main workflow used during training to derive attribution-guided penalties from segmentation errors. During training, an XAI method generates attribution maps from the segmentation network and utilizes them to quantitatively assess the model’s spatial attention. Regions corresponding to false negatives (FNs) that exhibit low attribution are interpreted as under-attended lesions, whereas false positives (FPs) with high attribution are regarded as manifestations of over-attention to healthy tissue. These two attention-related error modalities are transformed into complementary regularization terms and combined with the standard segmentation loss, thereby defining a training objective that simultaneously optimizes predictive accuracy and the alignment between the model’s attention and clinically relevant regions. Panel **(b)** overview of the proposed RGA framework during inference. Only the trained segmentation network is employed to generate lesion masks, while all XAI-based computations and RGA regularization are deactivated, thereby incurring no additional computational overhead at inference time. **(a)** Training stage. **(b)** Inference stage.

The objectives of this study are threefold. First, we investigate whether gradient-based attribution maps can be leveraged not only for post hoc interpretability, but also as an explicit supervisory signal during training for 3D MS lesion segmentation. Second, we examine whether this attribution-guided regularization enhances lesion sensitivity and lesion-wise detection performance across heterogeneous segmentation backbones, encompassing a CNN-based model, a transformer-based model, and a hybrid CNN—transformer architecture. Third, we evaluate whether such performance gains can be achieved without altering the inference-time architecture, thereby maintaining compatibility with computationally constrained deployment environments. The anticipated effect of RGA is a targeted reduction in missed lesions, potentially accompanied by a quantifiable precision–recall trade-off in certain configurations.

The remainder of this paper is structured as follows: Section 2 reviews the relevant literature; Section 3 details the datasets, preprocessing pipeline, and the proposed RGA framework; Section 4 reports and discusses the experimental findings; Section 5 draws conclusions and outlines future directions.

## 2 Related work

Clinically, manually segmenting MS lesions across serial MRI examinations is time-consuming, operator-dependent, and poorly scalable in long-term monitoring scenarios (5). The emergence of AI has opened new avenues for reproducible, high-throughput lesion quantification (23), with encoder-decoder architectures such as U-Net (24) and self-configuring frameworks such as nnU-Net (25) now representing robust baselines for clinical automation. A persistent gap remains, however: standard segmentation objectives tend to underdetect the smallest plaques, precisely those whose early identification carries the greatest prognostic and therapeutic relevance (12).

Generative and synthetic MRI methods represent a related strategy for addressing data scarcity and lesion-class imbalance in MS. Prior work has explored synthetic MRI for MS plaque detection (26) and, more recently, synthetic generation and denoising of paramagnetic rim lesions (27). These studies are closely connected to contrast-specific MS imaging, including phase-sensitive inversion recovery for improved intracortical lesion identification (28) and whole-brain Quantitative Susceptibility Mapping (QSM) processing (29). The main difference from our work is methodological and task-specific: these approaches modify or synthesize the image/contrast domain, often targeting plaque detection or paramagnetic rim lesion imaging, whereas our proposed framework does not generate additional images and is evaluated here as a training-time regularizer for voxel-wise lesion segmentation.

A considerable body of research has investigated the intrinsic class-imbalance problem inherent to MS lesion segmentation by reformulating or augmenting the underlying training objective functions. The standard Dice loss (10), while overlap-based and therefore more robust to imbalance than Cross-Entropy alone, assigns equal weight to all lesion voxels irrespective of lesion size, which systematically disadvantages the smallest plaques. Generalized Dice loss (12) partially corrects this by weighting class contributions inversely proportional to their volume, yet still operates purely at the prediction-output level. Focal loss (30), initially introduced for dense object detection in natural images, attenuates the contribution of well-classified voxels and thereby concentrates the gradient signal on more challenging negative examples. Owing to these properties, it has subsequently been widely incorporated into medical image segmentation frameworks (25). Tversky loss (31) extends the Dice coefficient by incorporating asymmetric penalization of false negatives and false positives, thereby enabling explicit control over the trade–off between recall and precision, an attribute of particular clinical relevance in lesion detection tasks. The Focal Tversky loss (32) further combines the focusing mechanism of Focal loss with Tversky’s asymmetric penalization, yielding improved sensitivity to small structures. Despite the diversity of these formulations, all of them act exclusively at the level of the final prediction: they modulate which voxels contribute to the loss, but exert no direct influence on the network’s internal spatial attention. Misclassified regions may therefore still exhibit low internal activations, making missed lesions invisible to the model’s feature representations even after gradient updates.

In parallel with loss-function research, transformer-based architectures have substantially advanced the state of the art in volumetric medical image segmentation by enabling long-range spatial reasoning beyond the receptive-field limitations of convolutional networks. The ViT (20) models demonstrated that self-attention over non-overlapping patches can match or surpass convolutional models on image recognition tasks, motivating its adaptation to dense prediction. UNETR (33) directly adopts a pure ViT encoder coupled with a CNN decoder in a U-shaped architecture, producing multi-scale feature maps suitable for volumetric segmentation. TransBTS (34) proposes a hybrid strategy in which convolutional layers extract local features before transformer modules integrate global context, reducing the token sequence length and computational cost relative to full-volume self-attention. Swin-Unet (35) introduces shifted-window attention within a U-Net topology, achieving linear complexity with respect to volume size while retaining hierarchical feature learning. These architectures have been evaluated on the MSLesSeg benchmark (36), confirming that global context modeling improves lesion delineation. However, the multi-head self-attention mechanism substantially increases model opacity relative to conventional CNNs, amplifying the interpretability deficit and motivating the integration of XAI tools to audit and regularize the learned spatial focus.

To support the clinical translation of automated methods, Guarnera et al. introduced the *MSLesSeg* dataset (36), a multi-center benchmark with neuroradiologist-validated ground truth encompassing three complementary MRI modalities: T1-weighted (T1-w), T2-weighted (T2-w), and Fluid-Attenuated Inversion Recovery (FLAIR). Baseline evaluations demonstrated that state-of-the-art architectures, including both diffusion- and transformer-based models, exhibit good concordance with expert delineations, achieving Dice Similarity Coefficients (DSCs) up to 0.685. Nevertheless, these models still show a substantial incidence of missed small lesions, indicating that a considerable clinical performance gap persists.

The dataset was subsequently employed as the official benchmark for the ICPR 2024 Competition on MS Lesion Segmentation (37). The top-performing entries (DSC up to 0.715) achieved incremental gains over the published baseline through elaborate multi-model ensembles and heuristic post-processing, a level of architectural complexity that is impractical for routine clinical deployment and largely opaque to clinical interpretation.

A complementary public resource is the 3D-MR-MS dataset introduced by Lesjak et al. (38), which provides multimodal MRI scans from 30 MS patients, along with consensus white matter lesion annotations from three expert raters. Tran et al. (39) used T1-w and T2-FLAIR inputs to validate WHASA-3D, a non-DL segmentation method, with a 10-subject optimization set and three 20-subject validation folds, reporting DSC values of 0.58 for WHASA-3D and up to 0.63 for optimized competing methods. More recently, De Rosa et al. (40) used T1-w and FLAIR inputs and evaluated pretrained lesion-segmentation algorithms on the same cohort as an external, data-blind test set without retraining, reporting DSC values in the 0.60–0.71 range.

Independent of segmentation advances, the medical imaging community has invested considerably in making deep learning models interpretable. Gradient- and activation-based XAI methods such as Grad-CAM (41), LayerCAM (42), and Integrated Gradients (IG) (43) generate dense spatial attribution maps that highlight the image regions driving a network’s prediction. A comprehensive review by Salahuddin et al. (19) surveyed the application of these tools to medical imaging, documenting their utility for model auditing, failure analysis, and building clinician trust in automated outputs. In MS specifically, post-hoc attribution analysis has been employed to visualize which lesion regions are captured or missed by segmentation networks, providing qualitative evidence that FN errors correlate with attenuated internal activations. However, all these applications treat XAI as a passive diagnostic instrument: attribution maps are computed after training or inference to inspect a frozen model, without feeding any corrective signal back into the optimization process.

A nascent line of research has begun leveraging XAI attribution maps as active, training-time supervisory signals for image classification. Weber et al., in their review (15), demonstrated that penalizing attention on non-discriminative regions during training improves both accuracy and spatial focus in classification models. A common thread across these works is their restriction to 2D classification tasks. Integrating XAI into the training loop of 3D volumetric segmentation networks, where attribution must be computed at every spatial location across high-resolution patches, introduces computational and architectural challenges that remain unaddressed in the existing literature. The RGA framework proposed in this work bridges this gap: it incorporates gradient-based attribution as a direct training signal within a 3D segmentation pipeline, penalizing both under-attention on missed lesions and over-attention on healthy tissue, while remaining architecturally agnostic and computationally tractable through periodic rather than per-step attribution. To ensure methodological rigor, quantitative comparisons are restricted to the validated baselines of the dataset paper (36), where evaluation protocols are standardized and reproducible across all reported methods.

Table 1 provides a structured matrix that systematically contrasts RGA against the main categories of related work reviewed in this section. The table differentiates approaches along six methodological dimensions: the approach, the core operating mechanism, whether XAI attribution is used as a training-time supervisory signal, applicability to 3D volumetric segmentation, architecture-agnostic design, and additional computational cost incurred at inference time.

**Table 1:** Structured comparison of the proposed RGA framework with related approaches from the reviewed literature.

Approach	Core mechanism	XAI train	3D Seg.	Arch-agnostic	Inf. overhead
Dice + CE (10, 11)	Voxel-overlap and per-voxel error at prediction output	No	Yes	Yes	N.A.
Gen. Dice (12), Focal loss (30), Tversky (31), Focal Tversky (32)	Volume-weighted, focal, or asymmetric FN/FP rebalancing at output	No	Yes	Yes	N.A.
Generative/synthetic methods (26, 27)	Synthetic image or contrast generation for plaque detection	No	N.A.	N.A.	N.A.
Post-hoc XAI analysis (19)	Attribution maps for post-training model inspection	No	N.A.	N.A.	N.A.
Training-time XAI (2D) (15)	2D attention penalty for image classification	Yes	No	Limited	Yes
**RGA (this work)**	**3D dual FN+FP attention penalties for volumetric segmentation**	**Yes**	**Yes**	**Yes**	**Zero**

The rows cover output-level losses, imbalance-aware losses, generative/synthetic methods, post-hoc XAI analysis, training-time XAI regularization, and RGA (this work).

Bold text indicates the proposed RGA framework.

Taken together, the reviewed literature and the structured comparison in Table 1 highlight a gap between post-hoc interpretability, imbalance-aware loss design, and training-time control of model attention in 3D lesion segmentation. Existing loss functions can rebalance voxel-wise or overlap-based prediction errors, and post-hoc XAI methods can reveal where a trained model focuses, but neither directly uses attribution information to guide the optimization of a volumetric segmentation network. This gap motivates the methodological design of RGA, which introduces attribution-guided penalties during training while preserving the original inference architecture. The following section therefore details the datasets, validation protocol, segmentation backbones, and RGA implementation used to evaluate this attribution-guided regularization strategy.

## 3 Materials and methods

This section describes the datasets used, the image preprocessing pipeline designed to handle multi-center scanner heterogeneity, the segmentation networks, the validation protocol, and the RGA framework, which constitutes the methodological contribution of this study.

### 3.1 Datasets

The proposed method was evaluated on two datasets: MSLesSeg and 3D-MR-MS.

#### 3.1.1 MSLesSeg dataset

The MSLesSeg dataset (36) is a publicly available multi-center benchmark designed to reflect the conditions encountered in routine clinical practice.

The cohort comprises 115 3D MRI examinations from 75 MS patients recruited across multiple clinical sites and scanners, deliberately introducing the intensity heterogeneity and acquisition protocol variability that characterize real-world neuroimaging data. In particular, 50 patients contributed data at a single time point, 15 at two time points, 5 at three, and 5 at four time points. For each examination, three co-registered modalities are provided: T1-w, T2-w, and FLAIR sequences.

Binary lesion masks were manually delineated by expert neuroradiologists and cross-validated across all three modalities to adjudicate anatomically ambiguous cases. The training cohort comprises 53 patients (21 male, 32 female; mean age 38.46 ± 10.59 years); the independent test cohort comprises 22 patients (6 male, 16 female; mean age 36.39 ± 10.16 years). Table 2 provides a summary of the demographic and clinical characteristics of both cohorts.

**Table 2:** Patient characteristics for the training and test cohorts.

Set	# Patients	# M/F	MS type	Age	EDSS	Lesion volume
				Mean (SD)	Mean (SD)	Mean (SD)
Training	53	21/32	50 RRMS 3 SPMS	38.46 (±10.59)	1.9 (±1.73)	12.167 (±13.233)
Test	22	6/16	21 RRMS 1 SPMS	36.39 (±10.16)	1.79 (±1.70)	10.231 (±10.912)

The columns report the total number of patients (# Patients), the number of male and female patients (# M/F), the distribution of patients according to multiple sclerosis subtype (MS Type), and the mean and standard deviation (SD) of age, Expanded Disability Status Scale (EDSS) score, and lesion volume.

#### 3.1.2 3D-MR-MS dataset

To enable a more comprehensive evaluation of RGA behavior, we additionally incorporated a second publicly available dataset, the *3D MR image database of Multiple Sclerosis patients with white matter lesion segmentations* (3D-MR-MS), originally introduced by Lesjak et al. (38). The dataset includes cross-sectional brain MRI scans from 30 MS patients and reference white matter lesion masks generated via a multi-rater consensus protocol. Three expert raters independently segmented lesions using semi-automated contouring tools, then refined their delineations in joint consensus sessions to produce the final reference segmentations. The archive was obtained from the muschellij2/open_ms_data GitHub repository (44), which distributes T1-w, contrast-enhanced T1-w, T2-w, and FLAIR images, brain masks, raw images, and co-registered derivatives. In this study, we employed the co-registered and resampled subset and restricted our analysis to the imaging modalities common to the complementary MSLesSeg dataset, specifically FLAIR, T1-w, and T2-w MRI.

The cohort includes 30 patients (7 male, 23 female; mean age 39.27 ± 10.12 years). The mean EDSS score was 2.61 ± 1.88, computed over the 27 patients for whom EDSS was available. Table 3 summarizes the demographic and clinical characteristics of the 3D-MR-MS cohort used in this work.

**Table 3:** Patient characteristics for the 3D-MR-MS cohort.

Set	# Patients	# M/F	MS type	Age	EDSS	Lesion volume
				Mean (SD)	Mean (SD)	Mean (SD)
3D-MR-MS	30	7/23	24 RRMS	39.27 (±10.12)	2.61 (±1.88)	17.39 (±16.07)
			2 CIS			
			2 SPMS			
			1 PRMS			
			1 N/A			

The columns report the total number of patients (# Patients), the number of male and female patients (# M/F), the distribution of patients according to multiple sclerosis subtype (MS Type), and the mean and standard deviation (SD) of age, Expanded Disability Status Scale (EDSS) score, and lesion volume.

### 3.2 Preprocessing and network architectures

MRI scans from multi-center cohorts present substantial inter-scanner intensity variability that can degrade automated analysis if left uncorrected. Three segmentation architectures were evaluated: nnU-Net v2 (25), a self-configuring residual encoder-decoder; UNETR (33), a transformer-based architecture with a pure ViT encoder; and TransBTS (34), a hybrid CNN–transformer model originally proposed for brain tumor segmentation. Each was trained with its own preprocessing pipeline, faithfully reproduced from its original implementation, to ensure a fair and representative comparison.

For the nnU-Net backbone, the v2 self-configuring framework handled automated preprocessing: brain volumes were cropped to remove extracerebral tissue and resampled to a common resolution, followed by independent z-score normalization of each MRI modality, computed on brain-foreground voxels. UNETR and TransBTS operated directly on the raw NIfTI volumes without resampling, applying per-channel z-score normalization computed on non-zero voxels. Patch sampling differed between the two models: UNETR used a foreground-biased strategy (50% lesion-centered, 50% random) to address class imbalance, whereas TransBTS adopted uniform random cropping.

All architectures processed volumetric patches of 128×128×128 voxels, preserving the three-dimensional spatial relationships among lesion voxels, which are critical for accurate morphological assessment. Data augmentation further differed by model: nnU-Net employed its comprehensive automated augmentation pipeline; UNETR used random flipping, $90^{\circ }$ rotations, and mild intensity perturbations; TransBTS applied random flips with intensity scaling and shifting.

The RGA regularization module is architecturally agnostic and was integrated as a modular component into all evaluated network architectures. Specifically, it enhances the training objective of each backbone with attribution-guided penalty terms ($L_{\text{FN}}$ and $L_{\text{FP}}$), while leaving the corresponding data preprocessing pipelines unmodified.

### 3.3 Evaluation metrics

Segmentation quality was assessed against the neuroradiologist-validated ground truth using a set of complementary metrics reflecting distinct aspects of clinical relevance. At the voxel level: the Dice Similarity Coefficient (DSC), quantifying overall lesion burden agreement and serving as the primary indicator; the True Positive Rate (TPR), measuring detection sensitivity; and the Positive Predictive Value (PPV), measuring precision. At the individual lesion level, most informative for tracking discrete plaques across serial MRI examinations, the Lesion-wise True Positive Rate (LTPR) counts the fraction of distinct lesion instances correctly identified, while the Lesion-wise False Positive Rate (LFPR) quantifies spurious detections. Volumetric accuracy is captured by the Absolute Volume Difference (AVD), directly relevant to lesion burden quantification in therapy trials, and spatial boundary precision by the Average Symmetric Surface Distance (ASSD). Rigorous formalizations of these evaluation metrics are presented in (36).

### 3.4 Learning and validation strategy

Training and evaluation followed the same 5-fold Cross-Validation (CV) protocol established in the MSLesSeg benchmark (36). Specifically, starting from the training/test split described in Section 3.1.1, five distinct folds were defined for training: Fold 1 used patients 1–43 for training and patients 44–53 for validation. Fold 2 used patients 1–33 and 44–53 for training and patients 34–43 for validation. Fold 3 used patients 1–23 and 34–53 for training and patients 24–33 for validation. Fold 4 used patients 1–13 and 24–53 for training and patients 14–23 for validation. Fold 5 used patients 11–53 for training and patients 1–10 for validation. This partitioning scheme was implemented to ensure a clear and systematic separation between training and validation folds. Subsequent evaluations were performed exclusively on patients with identifiers from 54 to 77, and all results presented in this work pertain to this independent held-out test cohort.

For each cross-validation fold, the model was trained for 200 epochs, with 200 iterations executed per epoch. Network optimization employs a polynomial learning rate decay schedule that induces relatively large parameter updates during the initial training phase and progressively smaller, more refined adjustments as training approaches convergence. The optimal model checkpoint was selected by monitoring segmentation quality on the held-out validation patients via an exponential moving average of the Dice score. The five fold-specific models were then evaluated independently on the held-out test set, and final performance is reported as the mean across folds, an unbiased and conservative estimate of generalizability to unseen clinical data.

For the 3D-MR-MS experiments, in the absence of an officially defined held-out test set for this dataset under the current experimental configuration, performance was assessed using deterministic 5-fold patient-level cross-validation, with 6 validation patients assigned to each fold. Predictions for each validation fold were generated independently and compared against the corresponding training-derived reference labels. The resulting performance metrics are therefore interpreted as internal cross-validation outcomes rather than as an official evaluation on an external test set.

### 3.5 Regularization via gradient attribution framework

The RGA framework employs XAI-derived attribution maps to interrogate the model’s internal spatial attention during training, effectively addressing the question: *where is the network focusing?* For a missed lesion (FN), the corresponding anatomical region manifests low attribution, indicating that the network has failed to allocate sufficient attention to the pathological area. In contrast, for a spurious detection (FP), the implicated region exhibits anomalously elevated attribution despite representing healthy tissue. Rather than interpreting these phenomena solely as errors at the level of predictive outputs, RGA explicitly couples them to the model’s attentional state: two complementary regularization terms are introduced, an under-attention penalty that drives the network to increase focus on missed lesions, and an over-attention penalty that attenuates unwarranted activations in healthy regions.

The RGA signal is applied periodically every N batches rather than at every training step, preventing the auxiliary regularization from dominating the primary segmentation objective. The pipeline operates through three sequential stages: feature extraction from the decoder, attribution-modulated error coupling, and per-sample volumetric normalization that equalizes gradient contributions across patients with widely differing lesion loads.

During forward propagation, intermediate spatial feature maps A are obtained from the last stage of the decoder. A spatial attribution map S∈[0,1] is then derived from A by assigning the target foreground logit to individual spatial locations using a gradient-based XAI method. The resulting map highlights the volumetric regions most influential to lesion detection.

Let R denote the raw attribution map before normalization. Because raw gradient-based attributions can have method-dependent scales and signed values, they are converted into non-negative foreground-attention weights before being used in the RGA loss. For LayerCAM, positive foreground gradients are combined with the target-layer activations and aggregated over channels. For IG, signed layer attributions are aggregated over channels and passed through a ReLU operation, so that negative evidence for the lesion class does not contribute as positive attention. The resulting non-negative map $R^{+}$ is normalized independently for each sample in the batch. For LayerCAM, max normalization is used, $S_{b,i}=\mathrm{clip}(R_{b,i}^{+}/(\max_{j}R_{b,j}^{+}+\epsilon ),0,1)$. For IG, a per-sample 99th-percentile normalization is used before clipping, $S_{b,i}=\mathrm{clip}(R_{b,i}^{+}/(Q_{0.99}(R_{b}^{+})+\epsilon ),0,1)$, which reduces sensitivity to isolated attribution outliers. The normalized map is detached from the attribution computation graph, resized to the segmentation-output resolution when necessary, and used only as a fixed spatial weighting mask in $L_{\mathrm{FN}}$ and $L_{\mathrm{FP}}$.

Two error maps are defined on the basis of the network’s estimated foreground probability $\hat{\;p}$ and the binary ground-truth label y∈{0,1}:

1. the *soft FNs*, (${\text{FN}}_{\text{soft}}=y\cdot (1-\hat{\;p})$), elevated when the ground truth corresponds to a lesion, but the model assigns a low predicted probability to that lesion;
2. the *soft FPs*, (${\text{FP}}_{\text{soft}}=(1-y)\cdot \hat{\;p}$), high in instances where the reference label corresponds to background, yet the model assigns a high predictive probability to that class.

To specifically target failures of the attention mechanism, these error maps are spatially weighted by the attribution map S. Concretely, the framework introduces two regularization terms: an under-attention penalty ($L_{\text{FN}}$), which penalizes regions containing lesions where the network both fails to detect the lesion and exhibits low attribution, and an over-attention penalty ($L_{\text{FP}}$), which penalizes background regions where the network erroneously produces high attribution responses. To maintain stable gradient magnitudes across a wide range of lesion burdens and to prevent samples with extensive plaque from dominating the optimization at the batch level, these penalties are first aggregated and normalized per sample, and only then averaged over the relevant sample set: lesion-containing samples for $L_{\mathrm{FN}}$ and all batch samples for $L_{\mathrm{FP}}$. The resulting loss components are defined as follows:

$$L_{\text{FN}}=\frac{1}{\left|V\right|}\sum_{b\in V}\frac{\sum_{i}(1-S_{b,i})\cdot {\text{FN}}_{{\text{soft}}_{b,i}}}{\max(1,\|y_{b}{\|}_{1})}$$ (1)

$$L_{\text{FP}}=\frac{1}{B}\sum_{b=1}^{B}\frac{\sum_{i}S_{b,i}\cdot {\text{FP}}_{{\text{soft}}_{b,i}}}{\max(1,\sum_{i}{\hat{\;p}}_{b,i})}$$ (2)

Here, i indexes spatial voxel positions and B denotes the batch size. The set $V=\{b\in \{1,\ldots ,B\}\;:\;\|y_{b}{\|}_{1}>0\}$ denotes the subset of batch samples containing at least one ground-truth lesion. Therefore, $L_{\mathrm{FN}}$ is averaged only over lesion-containing samples, because false-negative under-attention is meaningful only when a reference lesion is present. If |V|=0, the false-negative term is set to zero in the implementation. In contrast, $L_{\mathrm{FP}}$ is evaluated over all B samples, since false-positive attention can occur also in patches without ground-truth lesions.

The overall training objective $L_{\text{total}}$ (Equation 3) integrates the conventional segmentation loss terms ($L_{\text{Dice}}$ and $L_{\text{CE}}$) with the RGA-based regularization components, modulated by a dynamically scheduled weighting factor λ(e). To formally capture the periodic update mechanism, the indicator function I(tmodN=0) is defined, which enables the regularization term exclusively at every N-th batch. Accordingly, the total loss is expressed as:

$$L_{\text{total}}=L_{\text{Dice}}+L_{\text{CE}}+I(t\mathrm{mod}N=0)\cdot \lambda (e)\cdot (L_{\text{FN}}+L_{\text{FP}})$$ (3)

The regularization coefficient λ is governed by a three-phase dynamic schedule designed to prevent the XAI-based penalty terms from destabilizing the initial stages of training:

1. *Warmup* (epochs 0 to $E_{s}-1$): $\lambda =\lambda_{\min}$. The network is optimized exclusively for fundamental anatomical structure segmentation and is trained with the conventional loss function.
2. *Square-root Ramp* (epochs $E_{s}$ to $E_{e}$): The parameter λ increases from $\lambda_{\min}$ to $\lambda_{\max}$ along a concave trajectory. This induces an early concentration of penalty escalation, thereby maximizing its influence during the phase when the polynomial learning rate remains comparatively high.
3. *Plateau* (epochs $\geq E_{e}$): $\lambda =\lambda_{\max}$. During the fine-tuning phase, the spatial regularization hyperparameter is configured to operate at its maximal strength.

The spatial attribution map S can be generated by any XAI method yielding a dense spatial attribution. To enable a rigorous cross-evaluation of both backbone architecture and attribution strategy, two gradient-based methods were applied to all three architectures: LayerCAM (42), a lightweight single-pass method that weights activations by their gradients, and Integrated Gradients (IG) (43), a path-integrated approach that attributes the foreground logit by integrating gradients along a linear interpolation from a zero baseline to the actual feature map, satisfying completeness and sensitivity axioms that instantaneous gradient methods do not guarantee. The complete training pipeline is formally described in Algorithm 1. A reusable implementation of the RGA loss and attribution helpers is publicly available in a versioned GitHub release; the repository contents and limitations are reported in the Code Availability section.

Algorithm 1 **RGA training procedure. Each epoch begins with the computation of the dynamic weight λ(e) via a three-phase scheduler. At every batch, $f_{\theta }$produces the lesion probability map $\hat{\;p}$ and decoder features *A*; the standard loss $L_{\mathrm{Dice}}+L_{\mathrm{CE}}$ is always computed. Every *N* batches, a gradient-based XAI method derives the attribution map *S* from *A*, which drives the under-attention penalty $L_{\mathrm{FN}}$ and the over-attention penalty $L_{\mathrm{FP}}$, added to the total loss weighted by λ. Attribution is restricted to training and adds no inference-time overhead.**

**Input:** Segmentation network $f_{\theta }$, training data D, penalty schedule ($\lambda_{\min}$, $\lambda_{\max}$, $E_{s}$,$E_{e}$), XAI update frequency N

**Output:** Trained network weights $\theta^{*}$

 1: **for** each epoch e **do**

 2:  $\lambda \leftarrow \mathrm{Scheduler}(e,\;\lambda_{\min},\lambda_{\max},E_{s},E_{e})$         ▹ dynamic λ weighting schedule

 3:  **for** each batch (x,y)∈D **do**

 4:   $\hat{\;p},\;A\leftarrow f_{\theta }(x)$            ▹ Lesion probability map and decoder features

 5:   $L_{\mathrm{total}}\leftarrow L_{\mathrm{Dice}}+L_{\mathrm{CE}}$              ▹ Standard segmentation loss

 6:   **if** batch index is a multiple of N **then**            ▹ Periodic XAI regularization

 7:    $S\leftarrow \mathrm{Normalize}(\mathrm{XAI}\text{-}\mathrm{Attr}(f_{\theta },\;x,\;A))$            ▹ Attribution map normalized to [0,1]

 8:    Compute $L_{\mathrm{FN}}$            ▹ Penalize missed lesions with low attribution (Equation 1)

 9:    Compute $L_{\mathrm{FP}}$            ▹ Penalize healthy regions with high attribution (Equation 2)

 10:    $L_{\mathrm{total}}\leftarrow L_{\mathrm{total}}+\lambda \cdot (L_{\mathrm{FN}}+L_{\mathrm{FP}})$            ▹ Total loss (Equation 3)

 11:   **end** **if**

 12:   Backpropagate $L_{\mathrm{total}}$ and update θ

 13:  **end** **for**

 14: **end** **for**

15: **return** $\theta^{*}$

The RGA hyperparameter selection strategy was dataset-specific. For MSLesSeg, hyperparameters were tuned once using a grid search performed solely on fold 1. The optimal configuration was selected based only on the internal validation subset of fold 1, without access to the independent test set. After this selection step, the same configuration was fixed and used to train the models across all five MSLesSeg folds. This strategy preserves the independence of the MSLesSeg test set, although, as discussed in the Limitations section, it remains less stringent than a fully nested model-selection procedure.

For 3D-MR-MS, no official independent held-out test set was available under the adopted experimental configuration. Therefore, RGA hyperparameters were selected within the 5-fold cross-validation procedure using a nested-light strategy. For each outer fold, a compact internal screening was performed using only patients from the corresponding outer-training split; the held-out outer-validation fold used for performance reporting was never used for hyperparameter selection. The best configuration from this internal screening was then used to train the final model for that outer fold.

The hyperparameter domains explored for RGA model selection are summarized in Table 4.

**Table 4:** Hyperparameter grid-search domain for the RGA framework.

Parameter	Description	Search space
$\lambda_{\min}$	Initial weight	{0.01, 0.05}
$\lambda_{\max}$	Maximum weight	{1.0, 1.5,}
$E_{s}$	Epoch to start λ ramp	{5, 20}
$E_{e}$	Epoch to end λ ramp	{180, 200}
N	Update frequency	{4, 8}

The complete methodological workflow adopted in this study is summarized in Figure 2. Starting from the dataset-specific preprocessing and patient-level fold definition, each backbone is trained either with the standard segmentation objective or with the additional RGA terms derived from LayerCAM or IG attribution maps. Attribution maps are used only during training to compute the FN and FP attention penalties, whereas inference is performed with the unmodified segmentation backbone. The final predictions are then evaluated on the corresponding held-out MSLesSeg test set or 3D-MR-MS validation fold using the same quantitative metrics reported in the Results section.

**Figure 2:**
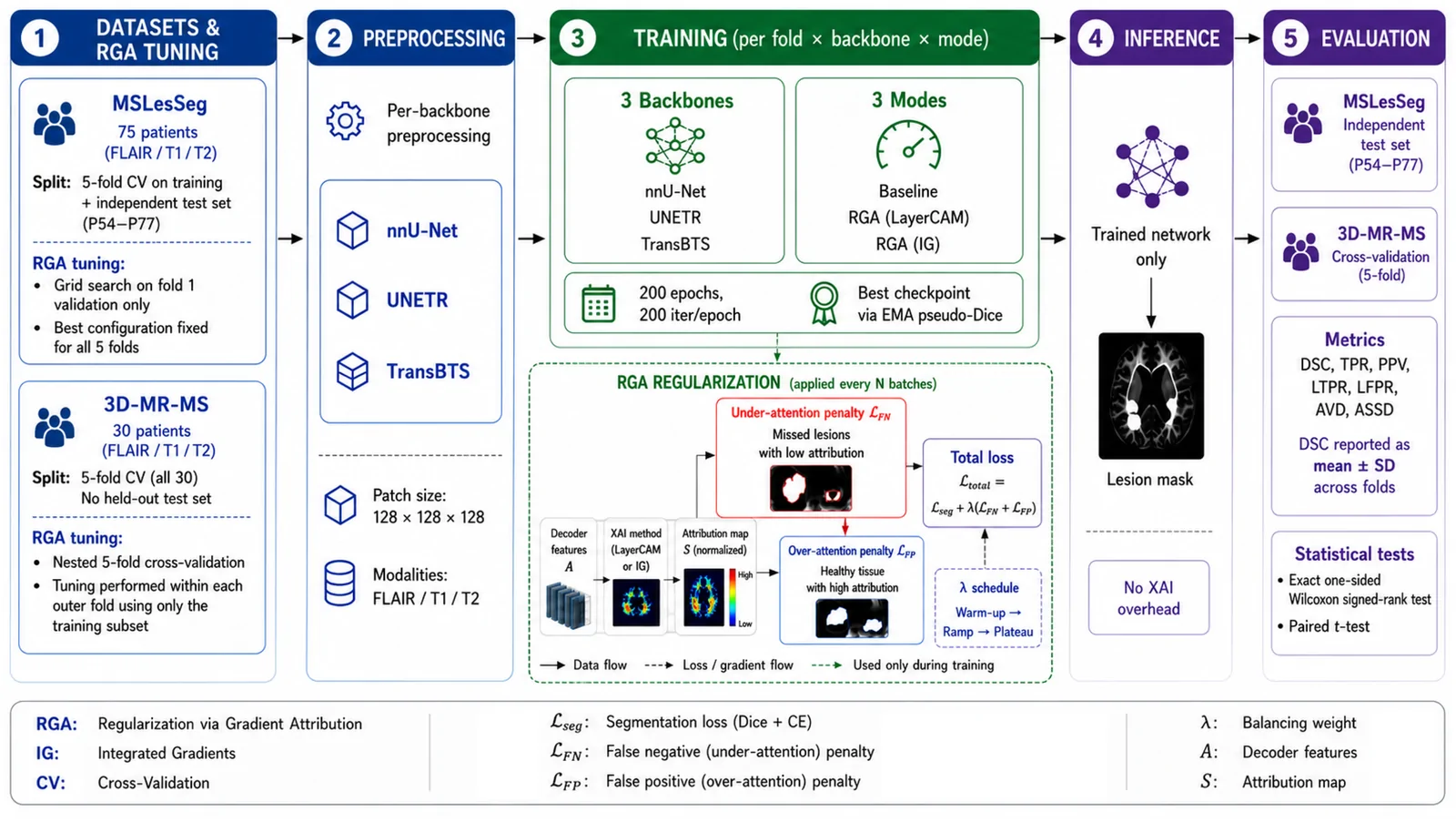
Complete methodological workflow of the proposed RGA framework. The diagram delineates the end-to-end pipeline, encompassing MRI data preprocessing and cross-validation partitioning, backbone-specific network training, attribution-guided regularization, attribution-free inference, and subsequent performance evaluation.

### 3.6 Conceptual positioning of RGA within IoMT workflows

To clarify the relationship between the proposed framework and Internet of Medical Things (IoMT) infrastructures, this subsection provides a dedicated conceptual description. Within an IoMT-oriented diagnostic pipeline, connected MRI scanners at peripheral clinical sites act as distributed sensing nodes that acquire volumetric brain examinations and transmit them, through secured health-platform networks, to processing nodes responsible for automated analysis. The segmentation model trained with the RGA framework is intended to operate as one such processing node, producing lesion masks and quantitative lesion-burden reports returned to the clinical workflow for radiological review and longitudinal monitoring.

The specific contribution of RGA is confined to the training stage, performed offline on dedicated computational infrastructure before deployment. During training, RGA uses gradient-based attribution maps to regularize the network’s spatial attention toward lesion regions; at inference time, all attribution computation and regularization terms are deactivated, and the deployed model reduces to the original segmentation backbone. The trained network therefore retains the exact inference-time architecture and computational footprint of its baseline counterpart, a property of direct relevance for IoMT edge scenarios in which inference must run on resource-constrained devices. RGA does not itself implement the data-acquisition, transmission, or orchestration components of an IoMT infrastructure; rather, it improves the reliability of the segmentation node that can be embedded within such an infrastructure.

As a concrete worked example, consider a peripheral clinical site acquiring a FLAIR/T1-w/T2-w MRI examination of an MS patient. The examination is transmitted through the IoMT platform to a processing node, where the RGA-trained segmentation network produces a lesion mask in a single forward pass without any attribution computation. The resulting lesion count, volume, and spatial report are returned to the clinical workflow to support disease-monitoring decisions. In this scenario, RGA contributes exclusively during the offline training of the segmentation node, while the IoMT infrastructure provides the acquisition, transmission, and orchestration layer that enables remote, asynchronous analysis.

The present study evaluates segmentation quality on the MSLesSeg benchmark and does not implement or benchmark an end-to-end IoMT deployment, as further discussed in the Limitations subsection. The relationship described above is therefore a statement of methodological compatibility and intended deployment positioning, rather than evidence of a validated IoMT system.

## 4 Results and discussion

The results and subsequent discussion are reported for two datasets: MSLesSeg and 3D-MR-MS.

### 4.1 MSLesSeg

Table 5 presents the segmentation performance obtained from 5-fold CV on the test set hold-out of three backbone architectures (nnU-Net, UNETR, TransBTS) and two XAI attribution methods (LayerCAM and IG), including two external reference baselines from (36) for comparative purposes. These external baselines are cited as reference values from the dataset paper; the primary evidence for the effect of RGA derives from the within-backbone comparisons between each baseline and its RGA-enhanced counterpart reported in the same table. Each architecture is analyzed in three configurations: without RGA, with RGA combined with LayerCAM, and with RGA combined with IG. This design enables a joint investigation of RGA’s regularization effect and the impact of the chosen attribution strategy across distinct network topologies.

**Table 5:** Quantitative segmentation performance on the MSLesSeg test set.

Model	DSC↑	TPR↑	PPV↑	LTPR↑	LFPR↓	AVD↓	ASSD↓
MSSegDiff (36)	0.6851	0.6719	0.7241	0.6250	0.2226	24.4500	3.4740
SwinUNETR (36)	0.6786	0.6676	0.7157	0.7017	0.2957	25.1200	3.6220
nnU-Net (baseline)	0.6979	0.6939	**0.7291**	0.7223	0.2460	37.0400	2.9600
nnU-Net (Focal Tversky)	0.7008	0.7254	0.6874	0.7437	0.2633	35.0841	2.7583
nnU-Net + RGA (LayerCAM)	0.7030	0.7365	0.6943	**0.7547**	0.2560	30.2000	2.7170
nnU-Net + RGA (IG)	**0.7056**	**0.7377**	0.6981	0.7522	**0.2430**	**29.0542**	**2.7004**
UNETR (baseline)	0.5884	0.6344	0.5898	0.6529	0.5695	60.7103	6.0329
UNETR + RGA (LayerCAM)	0.5953	**0.6501**	0.5850	**0.6622**	0.5881	**58.9352**	5.4591
UNETR + RGA (IG)	**0.6058**	0.6273	**0.6318**	0.6471	**0.5126**	69.9620	**4.9389**
TransBTS (baseline)	0.6527	0.6546	**0.6790**	0.6671	0.3813	33.2762	3.7175
TransBTS + RGA (LayerCAM)	0.6654	0.6973	0.6578	0.7027	0.4289	57.3864	3.4696
TransBTS + RGA (IG)	**0.6694**	**0.7002**	0.6696	**0.7083**	**0.3631**	**29.4301**	**3.2597**

For each backbone architecture, three training configurations are evaluated: without RGA, with RGA employing LayerCAM, and with RGA employing IG. For nnU-Net, an additional baseline trained with Focal Tversky loss in place of DiceCE is also reported. For each metric and baseline, the optimal value is indicated in boldface.

Replacing DiceCE with Focal Tversky increased nnU-Net DSC from 0.6979 to 0.7008. Both RGA variants achieved higher DSC and a more favorable overall metric profile.

The results demonstrate that the RGA framework consistently enhances DSC, TPR, and LTPR across all three backbone architectures, indicating that the attribution-guided penalty effectively redirects network attention toward previously undetected lesions. Moreover, RGA (IG) achieves larger DSC improvements than RGA (LayerCAM) for all architectures. This performance difference can be ascribed to the higher attribution fidelity of the IG method. In contrast to LayerCAM, which depends on instantaneous gradients and is therefore more susceptible to noise in high-resolution 3D volumes, IG provides more stable and reliable relevance estimates.

A consistent observation is that RGA does not enhance PPV. Instead, PPV exhibits a slight decrease relative to the corresponding baseline models. This behavior is a consequence of the formulation of the RGA objective: $L_{\text{FN}}$ effectively lowers the detection threshold in order to recover previously missed micro-lesions, which unavoidably leads to a small number of additional false positives in ambiguous peri-lesional tissue and consequently reduces precision. This precision–recall trade-off is considered clinically acceptable, as a missed plaque directly biases the estimation of overall disease burden, whereas a spurious detection can typically be resolved during subsequent neuroradiological review.

The magnitude of this trade-off is configuration-dependent. For TransBTS + RGA (LayerCAM), LFPR increases from 0.3813 to 0.4289, corresponding to an absolute increase of 0.0476 and a relative increase of approximately 12.5%. This increase should be interpreted together with the concomitant sensitivity gains, with TPR increasing from 0.6546 to 0.6973 and LTPR from 0.6671 to 0.7027. Since LFPR is defined as the fraction of predicted lesion components that do not overlap the reference annotation, it quantifies the proportion of predicted lesion candidates that require review, rather than the absolute number of additional false-positive lesions per patient. Clinically, this indicates that the LayerCAM configuration shifts TransBTS toward a more sensitivity-oriented operating point. Importantly, this behavior is not inherent to all RGA configurations: TransBTS + RGA (IG) improves DSC, TPR, LTPR, AVD, and ASSD while reducing LFPR from 0.3813 to 0.3631. Thus, when false-positive burden is a major deployment constraint, the IG-based configuration provides a more favorable sensitivity–precision balance.

Compared against the external baselines of (36), nnU-Net + RGA (IG) surpasses both MSSegDiff (DSC = 0.6851) and SwinUNETR (DSC = 0.6786) with a single end-to-end model, without ensembling or post-processing.

The lower baseline DSC of UNETR compared to nnU-Net and TransBTS warrants discussion. UNETR was implemented with the MONAI architecture using standard ViT-Base settings and a preprocessing/training pipeline aligned with the reference implementation, including non-zero-voxel z-score normalization, foreground-biased patch sampling, spatial and intensity augmentations, AdamW optimization, warmup-cosine scheduling, and DiceCE loss. The pure ViT encoder represents the input through tokenized patches and global self-attention, an inductive bias that may be less suited to detecting small, sub-centimeter MS lesions compared to the local receptive fields of convolutional encoders (nnU-Net) or hybrid CNN-transformer designs (TransBTS). Despite this architectural limitation, RGA consistently improves UNETR DSC across both LayerCAM and IG configurations and reduces fold-level variance, demonstrating that the proposed attribution-guided regularization remains effective even when the underlying backbone is suboptimal for the task.

The UNETR results also show that the effect of RGA is not uniform across all secondary metrics. With LayerCAM, UNETR improves DSC, TPR, LTPR, AVD, and ASSD, but LFPR increases from 0.5695 to 0.5881, indicating that the higher lesion sensitivity is accompanied by a modest increase in lesion-wise false positives. With IG, UNETR achieves the best DSC, PPV, LFPR, and ASSD among the UNETR configurations, but AVD worsens from 60.7103 to 69.9620. This suggests that IG regularization improves spatial overlap and boundary agreement while producing a less accurate global lesion-volume estimate. This behavior is plausibly related to the pure ViT encoder of UNETR, which represents the volume through tokenized patches and global self-attention rather than through strongly local convolutional features. For small MS lesions, the resulting attribution maps can be more spatially diffuse or less tightly calibrated to lesion volume than those derived from convolutional or hybrid CNN-transformer backbones. We therefore interpret the UNETR results as a metric-specific sensitivity–precision and volume-calibration trade-off rather than as a uniform deterioration of the RGA mechanism.

To quantify variability over folds, Table 6 reports DSC values as the mean ± standard deviation across the five trained fold-specific models. Across all three backbones, RGA also reduced the fold-level DSC standard deviation relative to the corresponding baseline. This suggests more stable cross-validation performance, plausibly because the attribution-based penalty adds a spatial constraint on lesion-relevant regions.

**Table 6:** 5-fold cross-validation DSC (mean ± standard deviation) for the three backbone architectures, evaluated with and without the incorporation of the RGA framework, on the MSLesSeg test set.

Backbone	Baseline	+ LayerCAM (RGA)	+ IG (RGA)
nnU-Net	0.6979 ± 0.0205	0.7030 ± 0.0172	0.7056 ± 0.0158
UNETR	0.5884 ± 0.0195	0.5953 ± 0.0156	0.6058 ± 0.0114
TransBTS	0.6527 ± 0.0207	0.6654 ± 0.0141	0.6694 ± 0.0123

In Table 7, statistical comparisons were performed on fold-level DSC values by comparing each RGA-enhanced model with its corresponding baseline. Because the methodological hypothesis was directional, namely that RGA should improve DSC relative to the baseline, we used an exact one-sided Wilcoxon signed-rank test as the primary non-parametric paired test. However, only five paired fold-level observations were available for each comparison; under this small-sample setting, the exact Wilcoxon test has a coarse discrete resolution, yielding the same near-threshold value (p=0.0625) for each RGA-vs.-baseline comparison. Therefore, these statistical results should be interpreted as descriptive fold-level directional evidence rather than as a standalone proof of generalization. Paired t-tests were also reported as a secondary parametric analysis and yielded concordant directional results, with p-values ranging from 0.0237 to 0.0332.

**Table 7:** Statistical tests comparing RGA variants vs. baseline on fold-level DSC values (5-fold cross-validation) on the MSLesSeg test set.

Backbone	RGA variant	Exact one-sided Wilcoxon p	Paired t-test p
nnU-Net	LayerCAM	0.0625	0.0332
	IG	0.0625	0.0299
UNETR	LayerCAM	0.0625	0.0309
	IG	0.0625	0.0237
TransBTS	LayerCAM	0.0625	0.0247
	IG	0.0625	0.0243

The exact one-sided Wilcoxon signed-rank test was used as the primary non-parametric paired test under the directional hypothesis that RGA improves DSC over baseline. Paired t-test results are reported as a secondary parametric paired analysis.

Model-size efficiency was further evaluated by normalizing DSC by the number of trainable parameters, as reported in Table 8. Because RGA is implemented as an additional loss term driven by XAI-derived attribution maps, it alters the optimization signal but not the segmentation backbone’s architecture. Consequently, for each backbone, the baseline, RGA (LayerCAM), and RGA (IG) configurations share the same number of parameters. Differences in DSC per million trainable parameters therefore reflect changes in segmentation performance rather than increased model capacity. This normalized measure is intended as a descriptive complement to the primary voxel-wise, lesion-wise, volumetric, and boundary metrics reported in Table 5.

**Table 8:** Descriptive parameter-efficiency view for baseline and RGA configurations on the MSLesSeg test set.

Backbone	Configuration	Params (M)	DSC	DSC per $10^{6}$ params
nnU-Net	Baseline	102.35	0.6979	0.0068
	RGA (LayerCAM)	102.35	0.7030	0.0069
	RGA (IG)	102.35	0.7056	0.0069
UNETR	Baseline	127.64	0.5884	0.0046
	RGA (LayerCAM)	127.64	0.5953	0.0047
	RGA (IG)	127.64	0.6058	0.0047
TransBTS	Baseline	32.92	0.6527	0.0198
	RGA (LayerCAM)	32.92	0.6654	0.0202
	RGA (IG)	32.92	0.6694	0.0203

DSC per million trainable parameters was computed as $\mathrm{DSC}/(\#\mathrm{params}/10^{6})$ and is reported as a secondary model-size indicator.

To quantify the training-time computational cost of the RGA framework, we measured the per-epoch training time for all three backbone architectures across the three training modes (baseline, RGA with LayerCAM, and RGA with IG) on the MSLesSeg dataset, fold 0. Each epoch comprised 200 iterations; a complete training run consists of 200 such epochs. The reported per-epoch times represent the mean ± standard deviation across the measured training epochs (Figure 3). The attribution update frequency N is one of the RGA hyperparameters explored during the grid search (Table 4), with candidate values N∈{4,8}. The benchmark presented here was performed with N=4, which represents the worst-case scenario in terms of training time: with N=4, the XAI-based regularization terms $L_{\mathrm{FN}}$ and $L_{\mathrm{FP}}$ are computed on 25% of the batches in each epoch. Using N=8 would halve the number of batches on which attribution is computed, proportionally reducing the IG overhead while maintaining the regularization benefit. LayerCAM-based RGA introduces negligible training overhead (approximately +2% to +7%) because it requires a single backward-pass gradient computation through the decoder. IG-based RGA incurs a larger overhead (+24% nnU-Net, +20% UNETR, +46% TransBTS) because IG is a path-integrated attribution method that interpolates from a baseline reference to the input, requiring several forward/backward passes per attribution computation. The overhead varies across backbones due to differences in the ratio between baseline forward-pass cost and additional gradient-computation cost: nnU-Net benefits from highly optimized mixed-precision training and I/O, UNETR uses an efficient transformer encoder, and TransBTS, with its hybrid CNN–transformer design, incurs a proportionally larger cost for additional gradient operations. The overhead is entirely confined to the training phase; inference-time architecture is unchanged.

**Figure 3:**
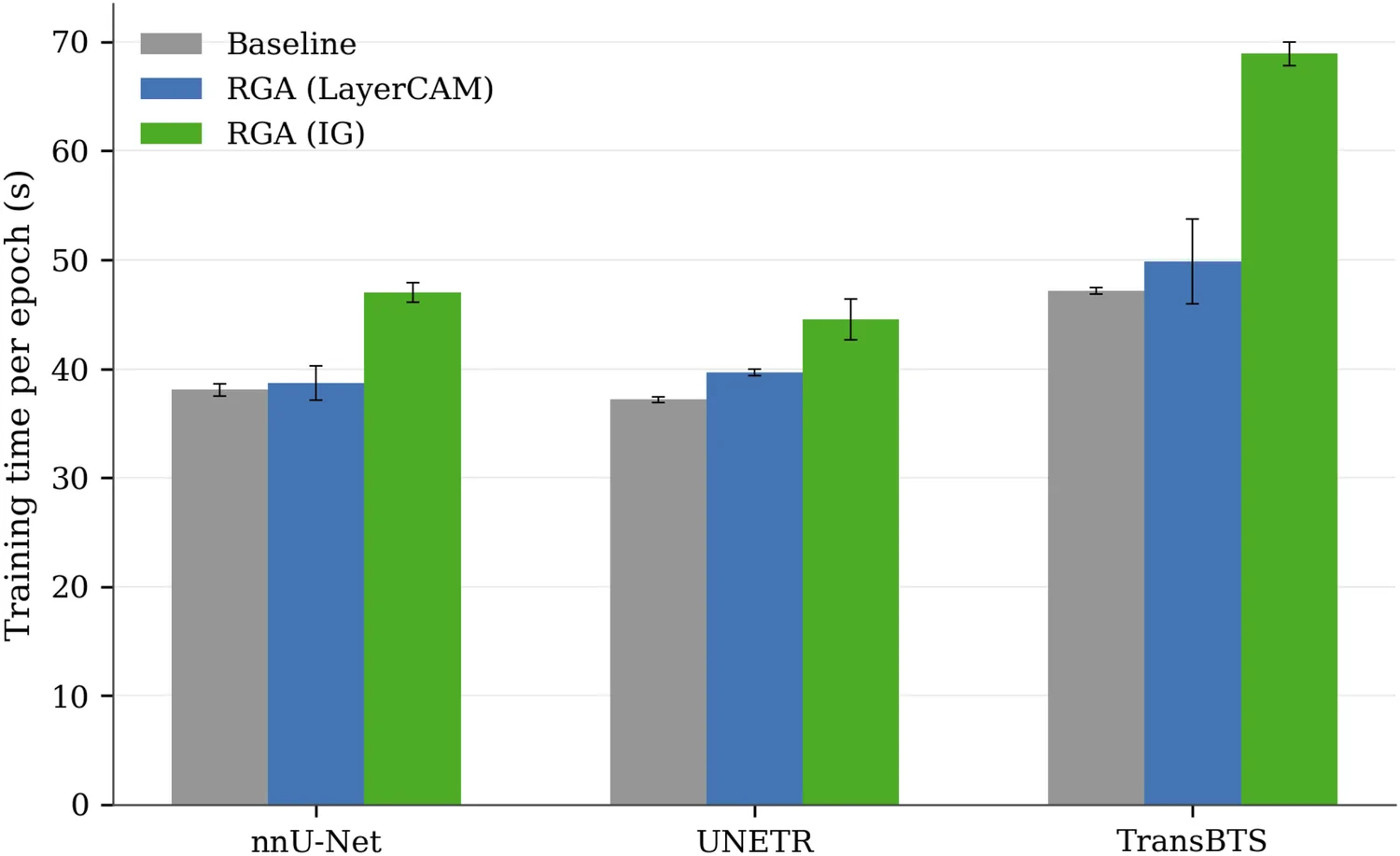
Per-epoch training time (mean ± standard deviation) for the three backbone architectures and three training configurations, measured on the MSLesSeg dataset.

#### 4.1.1 Ablation studies on the RGA loss

We evaluated 15 configurations with nnU-Net/LayerCAM on MSLesSeg fold 1 and the independent 22-patient test set, using the same 200-epoch protocol. The study compares DiceCE baseline, the individual RGA components, opposite FN/FP timings, fixed and scheduled weights, selected $\lambda_{\max}/N$ combinations, and complete RGA (Figure 4). Here, N is the attribution-update interval, i.e., RGA is computed every N batches. Complete RGA uses both $L_{\mathrm{FN}}$ and $L_{\mathrm{FP}}$, with a square-root ramp from 0.05 to 1.5 over epochs 5–199 and N=4. Owing to its concave shape, this schedule increases the weight earlier than a linear ramp.

**Figure 4:**
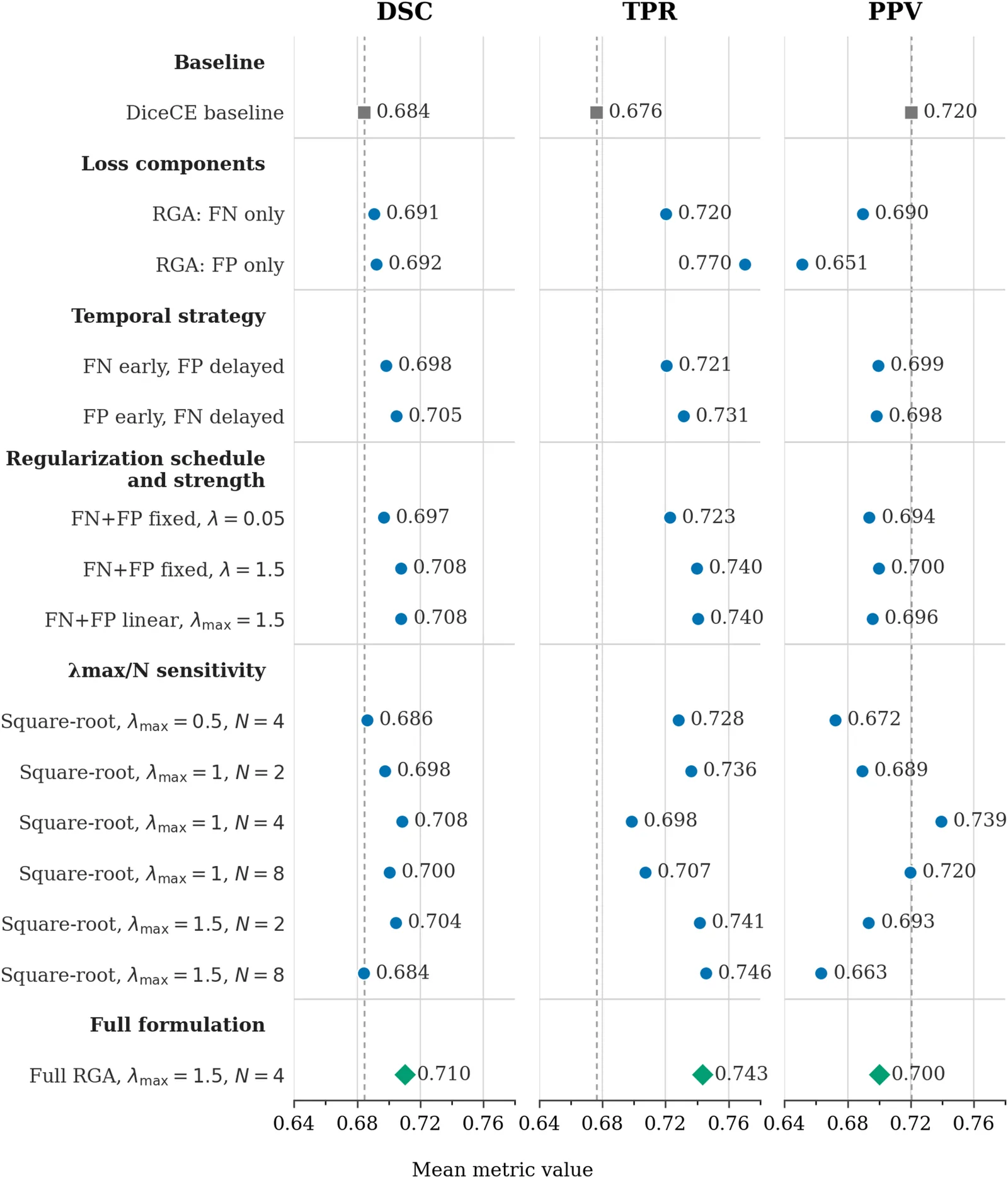
Targeted RGA ablation on MSLesSeg fold 1 using the test set. The three aligned panels report mean DSC, TPR, and PPV for 15 configurations grouped by experimental question. Vertical dashed lines indicate the DiceCE baseline reference value in each panel. The gray square denotes the baseline, blue circles denote intermediate ablations, and the larger green diamond denotes the complete RGA formulation.

The component ablation retained the complete schedule for the active term: FN-only disabled $L_{\mathrm{FP}}$, whereas FP-only disabled $L_{\mathrm{FN}}$. Both improved DSC over DiceCE, supporting their complementary contribution, although FP-only favored TPR at the expense of PPV. The temporal variants used the same weight range, with “early” denoting epochs 5–180 and “delayed” epochs 20–199. Applying FP early and FN delayed achieved a higher DSC than the inverse timing (0.705 vs. 0.698), with essentially unchanged PPV.

The schedule/strength ablation applied both penalties using a weak fixed weight (0.05), the maximum fixed weight (1.5), or a linear 0.05–1.5 ramp over epochs 5–199. The maximum fixed and linear variants approached complete RGA, whereas the weak fixed penalty was less effective, indicating that both regularization strength and scheduling influence the result.

The $\lambda_{\max}/N$ analysis used a shared square-root ramp with $\lambda_{\min}=0.05$. At both $\lambda_{\max}=1.0$ and 1.5, N=4 provided the highest DSC; other frequencies sometimes increased TPR at the expense of PPV. Overall, complete RGA achieved the highest DSC (0.710 vs. 0.684 for DiceCE; +0.026). Since this descriptive ablation is limited to one fold, backbone, attribution method, and run per configuration, it does not establish general hyperparameter robustness.

#### 4.1.2 Qualitative evaluation

Figure 5 presents qualitative examples of segmentation outputs for representative test-set slices obtained with each of the three backbone architectures. In this setting, all models were trained exclusively on training fold 1, and TPs (green) denote voxels correctly classified as lesions, FNs (blue) denote lesion voxels that are not detected by the model, and FPs (red) denote healthy voxels that are incorrectly classified as lesions. Therefore, this figure should be interpreted as an illustrative qualitative example of the error patterns observed in one fold-specific model, rather than as a replacement for the quantitative 5-fold evaluation reported in Table 5. The cross-fold performance summary and statistical analysis remain the primary evidence for the reported improvements. Across all panels, the integration of RGA produces a visibly reduced extent of FN regions compared with the corresponding baseline models, with previously missed lesions manifesting as additional green regions that spatially coincide with the yellow ground-truth contours. These qualitative observations are consistent with the quantitative results reported in Table 5: RGA consistently decreases the number of missed lesions across all architectures while inducing only a minor increase in FP predictions.

**Figure 5:**
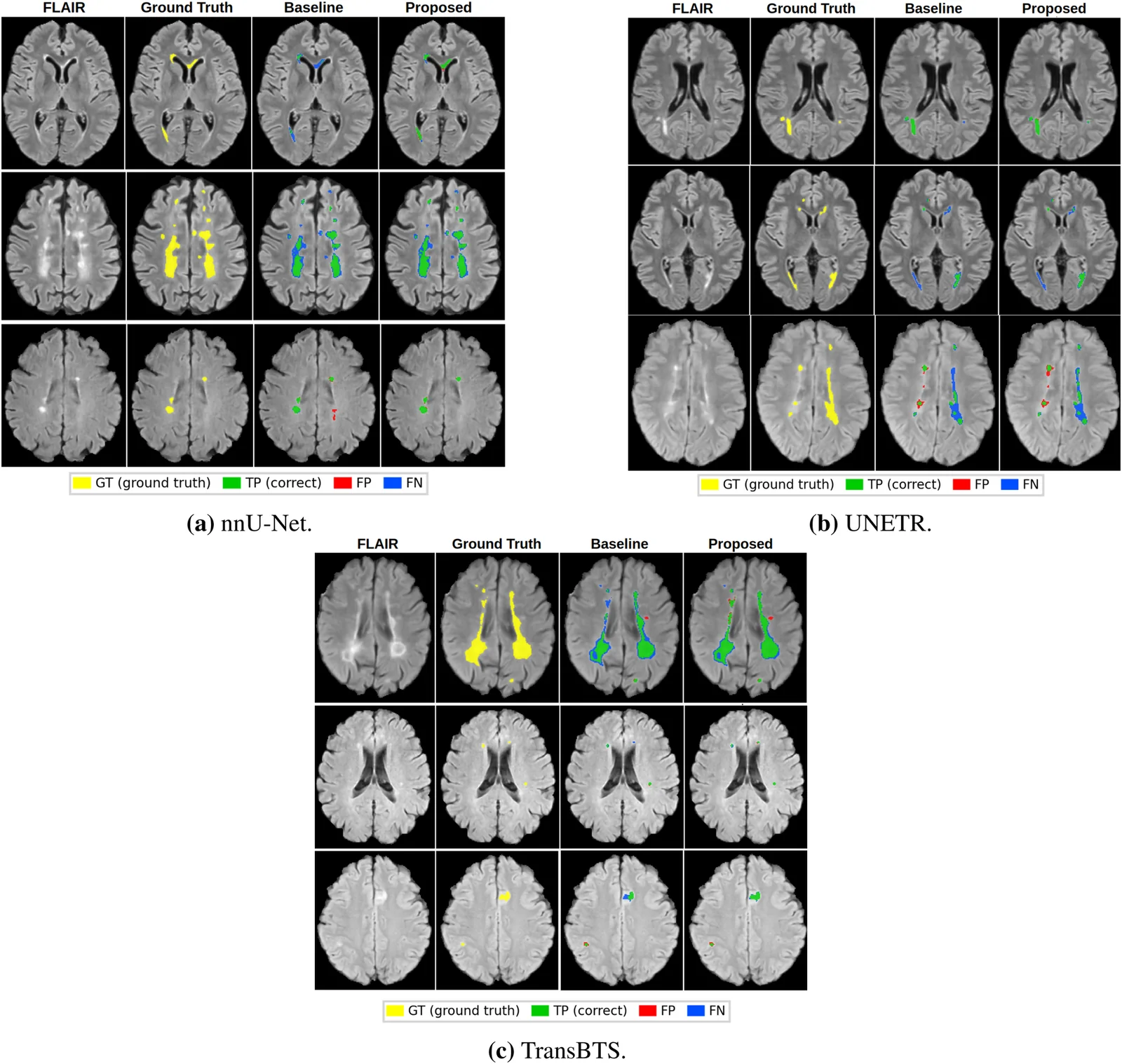
A qualitative comparison of MS lesion segmentations is shown for a representative axial slice from nine patients included in the MSLesSeg test set across three model architectures: **(a)** nnU-Net, **(b)** UNETR, and **(c)** TransBTS. Within each subfigure, the columns arranged from left to right depict: the input FLAIR MRI, the reference (ground-truth) annotation, the baseline model prediction (without RGA), and the prediction of the model incorporating RGA with IG. Segmentation results are color-coded as follows: ground truth (GT) in yellow, true positives (TPs) in green, false positives (FPs) in red, and false negatives (FNs) in blue.

Figure 6 provides a complementary patient-matched visualization that directly addresses the possible confounding effect of comparing different qualitative examples across architectures. By displaying the same three MSLesSeg test-set patients and the same axial slices for nnU-Net, UNETR, and TransBTS, the figure makes the differences between the baseline and RGA (IG) configurations easier to interpret. In several regions, RGA (IG) recovers additional lesion voxels, visible as green TP regions replacing blue FN regions in the corresponding baseline predictions. At the same time, some red FP regions remain or become more visible in selected cases, reflecting the expected sensitivity-precision trade-off also observed in the quantitative PPV and LFPR results. This additional figure is therefore intended as a qualitative, patient-aligned complement to the full cross-validation analysis rather than as a replacement for the quantitative evaluation.

**Figure 6:**
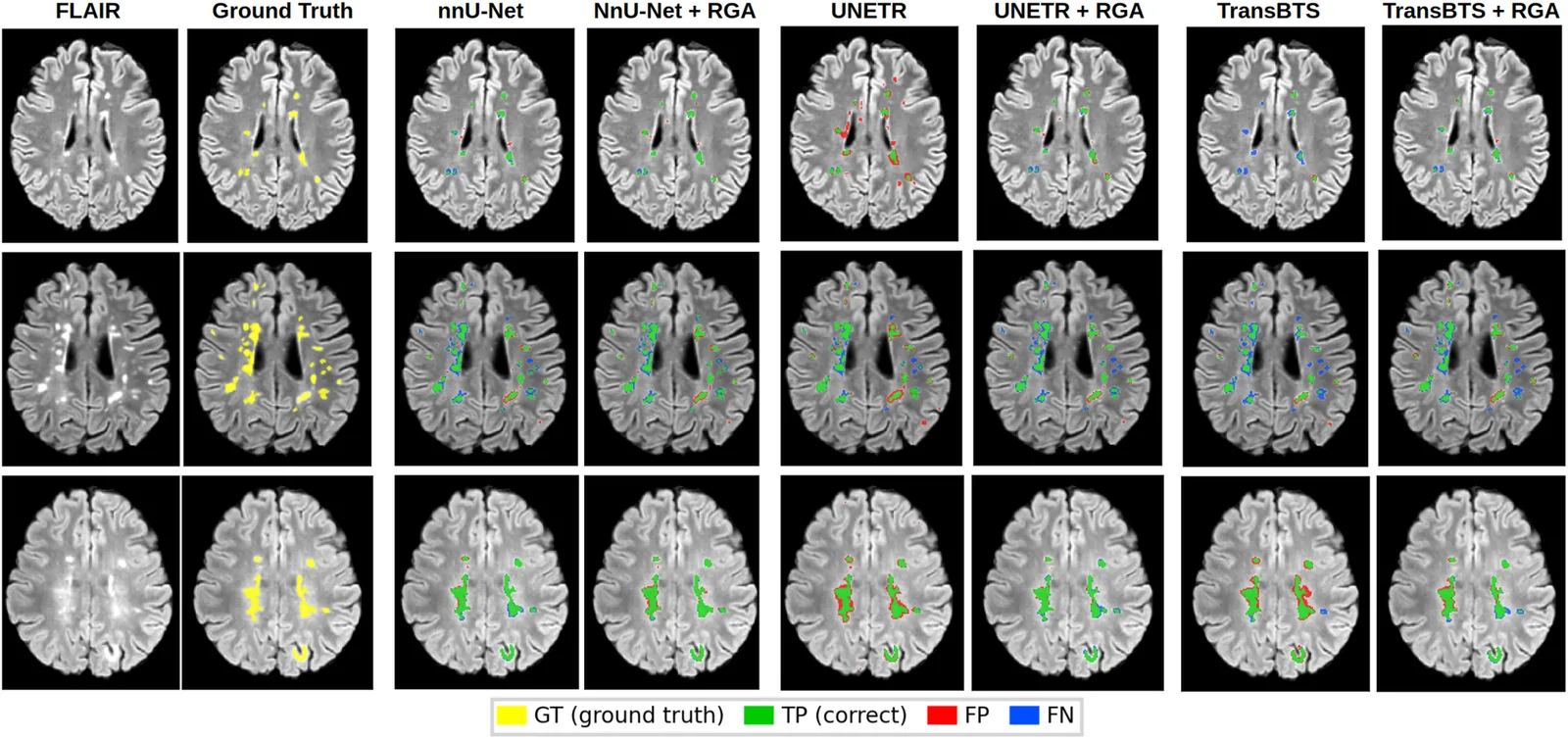
Patient-matched qualitative comparison on the same three MSLesSeg test-set patients across all backbone architectures. Each row corresponds to the same patient and axial slice for every model. Columns show, from left to right, the input FLAIR image, the reference ground-truth annotation, baseline nnU-Net, nnU-Net with RGA (IG), baseline UNETR, UNETR with RGA (IG), baseline TransBTS, and TransBTS with RGA (IG). Color coding is as follows: ground truth (GT) in yellow, true positives (TPs) in green, false positives (FPs) in red, and false negatives (FNs) in blue.

### 4.2 3D-MR-MS

Table 9 presents the segmentation performance obtained from 5-fold patient-level cross-validation on the 3D-MR-MS dataset. Overall, RGA reproduced the same direction observed on MSLesSeg. The IG-based variant achieved the highest DSC for all backbones, increasing performance from 0.7382 to 0.7466 for nnU-Net, from 0.6274 to 0.6366 for UNETR, and from 0.6906 to 0.7040 for TransBTS. These improvements were mainly accompanied by higher TPR and LTPR, indicating improved lesion recovery, while PPV, LFPR, and AVD showed a more heterogeneous behavior and therefore confirm the expected sensitivity–precision trade-off.

**Table 9:** Quantitative segmentation performance on the 3D-MR-MS dataset (5-fold cross-validation).

Model	DSC↑	TPR↑	PPV↑	LTPR↑	LFPR↓	AVD↓	ASSD↓
nnU-Net (baseline)	0.7382	0.7052	0.7936	0.5823	**0.2357**	**14.1176**	1.4502
nnU-Net + RGA (LayerCAM)	0.7432	0.7135	0.7933	0.5809	0.2506	14.3034	1.4309
nnU-Net + RGA (IG)	**0.7466**	**0.7180**	**0.7940**	**0.5850**	0.2450	14.2000	**1.4250**
UNETR (baseline)	0.6274	0.6204	0.7340	0.4782	0.4456	23.8918	2.1696
UNETR + RGA (LayerCAM)	0.6328	**0.6357**	0.7281	0.4850	0.4602	23.1900	1.9630
UNETR + RGA (IG)	**0.6366**	0.6330	**0.7350**	**0.4880**	**0.4320**	**22.7000**	**1.9500**
TransBTS (baseline)	0.6906	0.6977	**0.7698**	0.5339	0.3301	20.1565	1.9106
TransBTS + RGA (LayerCAM)	0.6984	0.7151	0.7621	0.5499	0.3466	20.5600	1.8150
TransBTS + RGA (IG)	**0.7040**	**0.7221**	0.7660	**0.5579**	**0.3218**	**19.5600**	**1.7380**

For each backbone architecture, three training configurations are evaluated: without RGA, with RGA employing LayerCAM, and with RGA employing IG. For each metric and baseline, the optimal value is indicated in boldface.

The fold-level summary in Table 10 further supports this pattern, since both RGA variants reduced the DSC standard deviation for all three architectures. The statistical tests in Table 11 should be interpreted descriptively, because only five paired fold-level observations were available for each comparison. Under this small-sample setting, the exact Wilcoxon signed-rank test has a coarse discrete resolution, and all comparisons yielded the same near-threshold value (p=0.0625). Therefore, these results should be viewed as directional fold-level evidence rather than as a standalone claim of statistical significance. The paired t-tests provide concordant support for the same trend, particularly for nnU-Net and TransBTS, while UNETR shows a weaker but consistent improvement pattern.

**Table 10:** 5-fold cross-validation DSC (mean ± standard deviation) for the three backbone architectures, evaluated with and without the incorporation of the RGA framework, on the 3D-MR-MS dataset.

Backbone	Baseline	+ LayerCAM (RGA)	+ IG (RGA)
nnU-Net	0.7382 ± 0.0759	0.7432 ± 0.0730	0.7466 ± 0.0720
UNETR	0.6274 ± 0.0646	0.6328 ± 0.0609	0.6366 ± 0.0584
TransBTS	0.6906 ± 0.0714	0.6984 ± 0.0677	0.7040 ± 0.0655

**Table 11:** Statistical tests comparing RGA variants vs. baseline on fold-level DSC values (5-fold cross-validation) on the 3D-MR-MS dataset.

Backbone	RGA variant	Exact one-sided Wilcoxon p	Paired t-test p
nnU-Net	LayerCAM	0.0625	0.0469
	IG	0.0625	0.0320
UNETR	LayerCAM	0.0625	0.0590
	IG	0.0625	0.0510
TransBTS	LayerCAM	0.0625	0.0337
	IG	0.0625	0.0276

#### 4.2.1 External data-blind validation

To directly assess cross-dataset generalization without cohort-specific retraining, we performed an additional data-blind external validation using the nnU-Net architecture. Five baseline models and five RGA-LayerCAM models were trained exclusively on the existing MSLesSeg folds for 200 epochs, with the best checkpoint selected from the corresponding MSLesSeg validation fold. Once frozen, each fold-specific model was evaluated on all 3D-MR-MS dataset. The external inputs were restricted to the three modalities shared by the datasets and reoriented to the coordinate convention used by the MSLesSeg pipeline; the source volumes were already co-registered and isotropic at 1 mm. No 3D-MR-MS case was used for training, checkpoint selection, hyperparameter tuning, or nnU-Net planning, and no probability ensemble was created. Table 12 reports the mean and standard deviation across the five fold-specific model results.

**Table 12:** Data-blind external validation of MSLesSeg-trained nnU-Net models on the 30-patient 3D-MR-MS cohort.

Metric	Baseline	RGA-LayerCAM	Δ LayerCAM–baseline	Favorable folds
DSC ↑	0.5200±0.0298	0.5557±0.0201	+0.0357	5/5
TPR ↑	0.4036±0.0371	0.4504±0.0297	+0.0468	5/5
PPV ↑	0.8071±0.0313	0.7960±0.0192	−0.0111	2/5
LTPR ↑	0.2361±0.0125	0.2498±0.0184	+0.0137	4/5
LFPR ↓	0.2202±0.0295	0.1726±0.0239	−0.0476	5/5
AVD ↓	(50.63±5.39)%	(44.88±4.64)%	−5.76 pp	5/5
ASSD ↓	6.276±0.667 mm	5.739±0.946 mm	−0.538 mm	4/5

Values are the mean ± standard deviation across the five fold-specific model means. The delta is RGA-LayerCAM minus baseline; “favorable folds” counts changes in the desired metric direction. AVD is reported as a percentage and ASSD in millimeters. Bold values in the delta column and in favorable folds indicate metrics where an improvement was consistently achieved across all 5/5 validation folds.

RGA-LayerCAM improved the DSC baseline from 0.5200 to 0.5557 and increased TPR by 0.0468, with favorable changes in all five fold-specific models. It also reduced LFPR by 0.0476 and AVD by 5.76 percentage points in all five folds, while PPV decreased modestly by 0.0111. LTPR and ASSD improved in four of the five models. The exact one-sided paired Wilcoxon test for the directional DSC hypothesis yielded p=0.03125; because only five fold-level pairs were available, this result should be interpreted as descriptive directional evidence rather than as standalone proof of generalization.

Despite this consistent relative benefit, the absolute external performance remained below the cohort-specific results obtained after retraining on 3D-MR-MS (Table 9). This gap indicates a substantial cross-dataset domain shift. RGA therefore attenuated the performance degradation and preserved its favorable direction without exposure to the external cohort, but it did not eliminate the distribution mismatch; adaptation or cohort-specific retraining may still be required to reach performance suitable for clinical use.

#### 4.2.2 Qualitative evaluation

Figure 7 provides a patient-matched qualitative visualization on the 3D-MR-MS dataset. By displaying the same three patients and the same axial slices for nnU-Net, UNETR, and TransBTS, the figure illustrates the qualitative behavior of RGA on an independent cohort. Consistent with the MSLesSeg observations, RGA (IG) recovers additional lesion voxels, visible as green TP regions replacing blue FN regions in the corresponding baseline predictions, while some red FP regions remain or become more visible in selected cases. This additional figure is intended as a qualitative, patient-aligned complement to the full cross-validation analysis.

**Figure 7:**
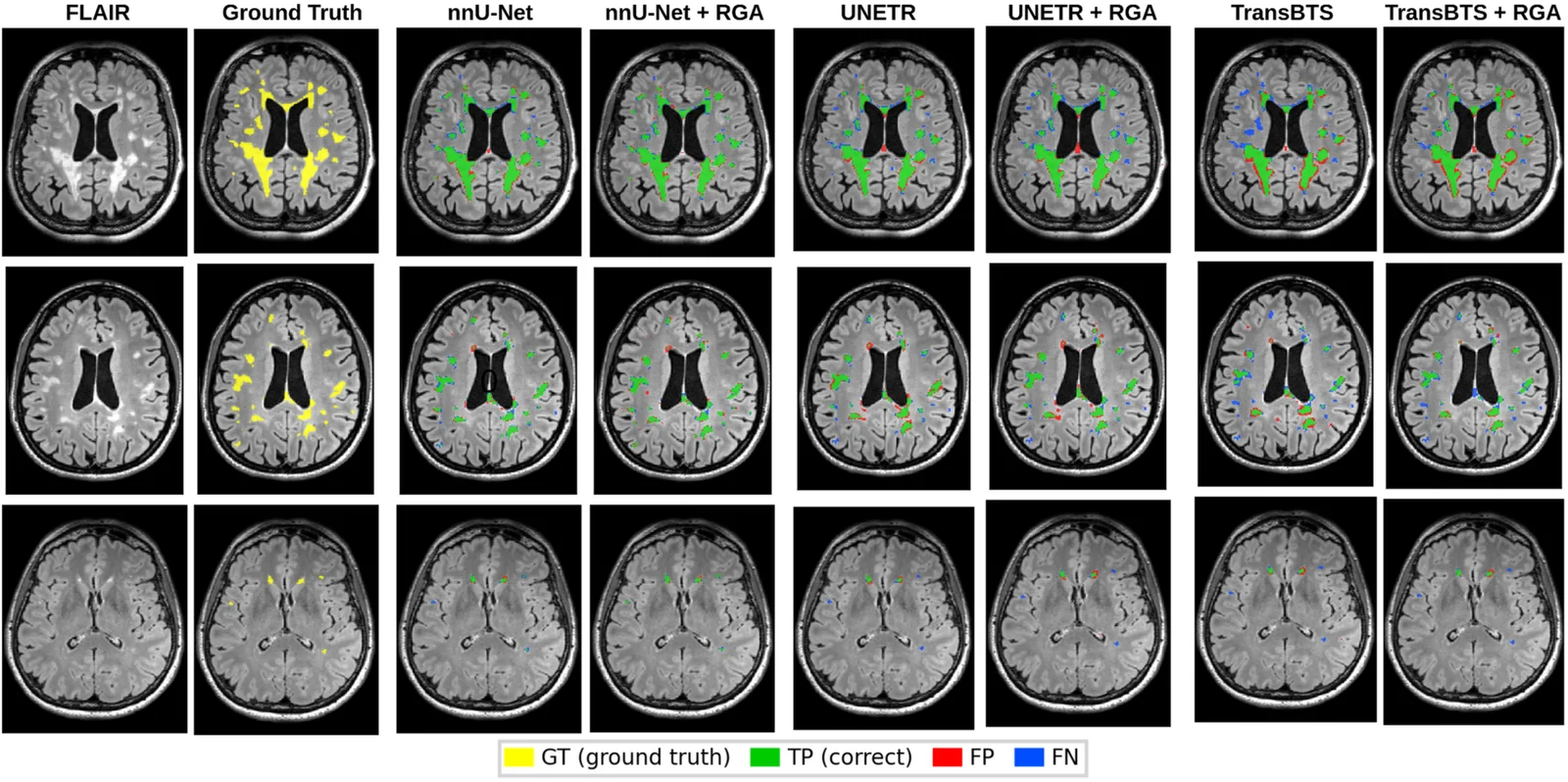
Patient-matched qualitative comparison on the same three 3D-MR-MS patients across all backbone architectures. Each row corresponds to the same patient and axial slice (in standard RAS convention) for every model. Columns show, from left to right, the input FLAIR image, the reference ground-truth annotation, baseline nnU-Net, nnU-Net with RGA (IG), baseline UNETR, UNETR with RGA (IG), baseline TransBTS, and TransBTS with RGA (IG). Color coding is as follows: ground truth (GT) in yellow, true positives (TPs) in green, false positives (FPs) in red, and false negatives (FNs) in blue.

### 4.3 Limitations

The present study has some limitations. We executed a direct imbalance-aware loss comparison by replacing DiceCE with Focal Tversky in nnU-Net and repeating the complete five-fold MSLesSeg evaluation. Focal Tversky improved the conventional baseline DSC but remained below both nnU-Net RGA configurations. This experiment is nevertheless restricted to one backbone, one dataset; it is not an exhaustive comparison across architectures, datasets, or combinations of RGA with imbalance-aware objectives.

We performed a targeted RGA ablation on MSLesSeg fold 1, examining the separate $L_{\mathrm{FN}}$ and $L_{\mathrm{FP}}$ terms, penalty timing, constant and scheduled regularization, and selected combinations of $\lambda_{\max}\in \{0.5,1.0,1.5\}$ and N∈{2,4,8}. This analysis remains restricted to one backbone, LayerCAM, one fold and a single run per configuration; it is not a complete factorial, multi-fold, multi-backbone, multi-attribution, or multi-seed evaluation. It should consequently be interpreted as descriptive component- and hyperparameter-level evidence rather than as a general robustness claim.

For the MSLesSeg experiments, the RGA hyperparameters were determined via a grid search on fold 1 and then held constant across all remaining cross-validation folds. This procedure ensured that the independent test set remained unseen during hyperparameter selection. However, it is methodologically less stringent than a fully nested model-selection framework and may introduce fold-selection bias, as the chosen configuration could be partially tailored to the specific validation characteristics of fold 1. Reporting fold-level means ± standard deviations, together with the additional 3D-MR-MS experiments employing nested hyperparameter optimization, partially mitigates this limitation but does not constitute a full substitute for a fully nested evaluation on MSLesSeg.

The additional data-blind MSLesSeg-to-3D-MR-MS experiment demonstrated a consistent relative benefit from RGA-LayerCAM, but its lower absolute performance compared with cohort-specific retraining revealed a substantial cross-dataset domain shift. Consequently, deployment on unseen cohorts may require site- or cohort-specific adaptation or retraining, and the present external results should not be interpreted as evidence that RGA alone removes scanner- and cohort-related distribution differences.

The present comparison does not include recently proposed parameter-efficient architectures, including the efficient transformer-based approaches (45, 46). Such architectures are highly relevant for IoMT-oriented and resource-constrained deployment scenarios, but a fair assessment would require adapting the full 3D segmentation pipeline, retraining across folds, and evaluating both segmentation accuracy and computational efficiency under the same protocol used for the other backbones.

Although RGA does not alter the inference architecture and therefore introduces no additional inference-time modules, we did not conduct a dedicated memory, latency, or edge hardware deployment benchmark. The IoMT discussion should therefore be interpreted as a statement of methodological compatibility and practical potential, rather than as evidence of a validated IoMT deployment.

Evaluating combinations with further imbalance-aware losses, extending the RGA ablation across folds, backbones, attribution methods, random seeds, and update frequencies, and performing deployment-oriented runtime and memory profiling represent important directions for future work.

## 5 Conclusion

This study introduces the Regularization via Gradient Attribution (RGA) framework, a module that exploits gradient-based XAI attribution maps as an explicit supervisory signal during optimization for 3D MS lesion segmentation. Instead of constraining the learning process solely at the level of the final prediction, RGA regularizes the network by penalizing cases where lesions exhibit low internal attribution and by suppressing spurious high-attribution responses in anatomically normal, non-lesional tissue. RGA was evaluated on two public MS lesion segmentation datasets, MSLesSeg and 3D-MR-MS, across three backbone architectures (nnU-Net, UNETR, and TransBTS) and two attribution methods (LayerCAM and Integrated Gradients). Across both datasets, RGA consistently improved DSC, TPR, and LTPR compared with the respective baselines, with the IG-based variant providing the most consistent gains. The additional 3D-MR-MS experiments reproduced the same overall direction observed in MSLesSeg, while remaining interpretable as internal cross-validation evidence, given the absence of an official independent test set. Qualitative assessment further supported these findings, showing a visually consistent reduction in missed lesion regions, with the expected sensitivity–precision trade-off in selected cases.

The properties exhibited by RGA indicate that it is a potentially suitable component for IoMT-oriented infrastructures. RGA introduces no additional computational overhead during the inference phase. The generation of attribution maps and the integration of gradient-based attribution regularization are confined exclusively to the training stage, thereby rendering RGA a computationally lightweight training-time augmentation for segmentation backbones that may be integrated into IoMT-oriented workflows after dedicated deployment validation.

Beyond the numerical improvements, the main implication of these results is that attribution maps can be used not only for post-hoc interpretation, but also as operational training signals for volumetric medical image segmentation. This provides a practical route toward models whose optimization is guided jointly by prediction accuracy and spatial attention behavior, without modifying the inference architecture.

The proposed framework should be interpreted in light of the aforementioned limitations. In particular, RGA was not experimentally compared against the full spectrum of class-imbalance–aware loss functions or with recently introduced parameter-efficient architectures. Moreover, the targeted component and $\lambda_{\max}/N$ sensitivity analyses were constrained to a single MSLesSeg fold, one backbone architecture, a single attribution method (LayerCAM), a set of selected rather than fully factorial hyperparameter combinations, and only one run per configuration. In addition, no benchmark on edge-device deployment was conducted.

Future work will therefore aim to: (i) integrate RGA with alternative segmentation loss functions; (ii) broaden the ablation study across multiple folds, network architectures, attribution methods, random seeds, and a denser factorial hyperparameter grid; (iii) systematically evaluate runtime and memory footprints in deployment-oriented scenarios; and (iv) extend the framework to longitudinal, multi-time-point MS cohorts as well as additional neuroimaging datasets.

## Data Availability

Publicly available datasets were analyzed in this study. This data can be found here: https://doi.org/10.6084/m9.figshare.27919209. The 3D-MR-MS dataset is available through the Open MS Data repository at https://github.com/muschellij2/open_ms_data. No new patient data were generated in this study. The reusable source implementation of RGA is publicly available in the versioned GitHub: https://github.com/salvatoregiugliano/rga-ms-lesion-segmentation. The release includes the RGA false-negative and false-positive penalties, saliency normalization and scheduling, LayerCAM and Integrated Gradients helpers, a model-independent integration wrapper, and a synthetic usage example.
